# LncRNA BCYRN1/miR-490-3p/POU3F2, served as a ceRNA network, is connected with worse survival rate of hepatocellular carcinoma patients and promotes tumor cell growth and metastasis

**DOI:** 10.1186/s12935-019-1081-x

**Published:** 2020-01-06

**Authors:** Shichao Ding, Yanfeng Jin, Qingzhi Hao, Yanmeng Kang, Ruiping Ma

**Affiliations:** 1 Affiliated Hospital of Shandong Academy of Medical Sciences, Shandong First Medical University, Internal Second Medicine, Jinan, Shandong China; 2 Department of Gastroenterology, Yantai Yuhuangding Hospital, Yantai, Shandong China; 3 Department of Peripheral Vascular Diseases, The Affiliated Hospital of University of Traditional Chinese Medicine, Jinan, Shandong China; 4 Department of Respiratory Diseases, The First Affiliated Hospital of Shandong First Medical University, Jinan, China; 5 Department of Respiratory Diseases, Shandong Provincial Qianfoshan Hospital, Shandong University, Jinan, Shandong China; 6 Department of Liver Diseases, The First Affiliated Hospital of Shandong First Medical University, Jinan, Shandong China; 7 Department of Liver Diseases, Shandong Provincial Qianfoshan Hospital, Shandong University, No. 16766, Jingshi Road, Jinan, 250000 Shandong China

**Keywords:** Hepatocellular carcinoma, BCYRN1, MiR-490-3p, POU3F2

## Abstract

**Backgrounds:**

LncRNA Brain Cytoplasmic RNA 1 (BCYRN1) has been certified to modulate cancer cells growth and aggressiveness in several tumors. However, research about function of BCYRN1 in hepatocellular carcinoma (HCC) is limited. Therefore, our research intends to explore the function of BCYRN1 in HCC.

**Methods:**

HepG2 and BEL-7402 cell lines were employed for later function experiments. Differently expression levels of BCYRN1, miR-490-3p, and POU class 3 homeobox 2 (POU3F2) were determined on the base of TCGA dataset including 375 HCC patients and 50 normal. 370 cases of patients, which have fairly complete clinical data, were utilized for survival analysis of BCYRN1, miR-490-3p, or POU3F2 by Kaplan–Meier method. Relative expression pattern of BCYRN1 was examined by quantitative real time polymerase chain reaction (qRT-PCR), and relative expression level of POU3F2 was assessed by qRT-PCR and western blot. Cell biological behaviors were analyzed by cell counting kit-8, cloning formation, and transwell assays. Bioinformatics software and dual luciferase assay were applied to predict and confirm the targeted relationship between BCYRN1 and miR-490-3p, as well as miR-490-3p and POU3F2. Further associations among BCYRN1, miR-490-3p, and POU3F2 were analyzed by rescue assays.

**Results:**

Our results exhibited that BCYRN1 was over expressed in HCC samples, which was connected with unfavorable prognosis in HCC patients. In addition, a series of experiments exhibited that overexpression of BCYRN1 significantly expedited HCC cells growth, clone formation, and movement abilities, and vice versa. Moreover, targeted relationships between BCYRN1 and miR-490-3p, as well as miR-490-3p and POU3F2 were affirmed by dual luciferase assay. Furthermore, POU3F2 expression was negatively connected with the expression of miR-490-3p and positively associated with BCYRN1 expression. Whilst, either overexpression of miR-490-3p or knockdown of POU3F2 could remarkably inhibit the increasing trends of proliferation, clone formation, invasion, and migration abilities induced by BCYRN1 in HCC cells.

**Conclusions:**

BCYRN1, served as a competing endogenous RNA, up-regulated the expression of POU3F2 to promote the development of HCC through sponging miR-490-3p, supplying novel molecular targets and underlying prognostic biomarkers for HCC therapy.

## Background

Hepatocellular carcinoma (HCC) is the most frequent malignant liver tumor type in the world, which is an important problem affecting human health [1]. Up until now, early diagnosis and effective therapy of HCC remain a challenge [2]. Although surgical resection is the preferential treatment for HCC patients [3], nearly 70% of HCC patients recurred within 5 years after hepatectomy [4], partly due to the absence of effective targeted therapy, resulting in low long-term survival after surgery [5]. Consequently, it is urgent to reveal the underlying molecular mechanisms of HCC and search for the target molecules for early diagnosis and prognosis.

In recent years, as more and more lncRNAs have been identified, their functions have received extensive attention. Among numerous lncRNAs, BCYRN1 has been reported to be a critical molecule to regulate cancer cells survival and proliferation [6]. For example, on the basis of published literature, BCYRN1 promoted cells metastasis of non-small-cell lung cancer through up-modulating MM9 and MMP13 [7]. In addition, study from Gu et al. has suggested that BCYRN1 regulated proliferation of colorectal cancer cells through up-regulating NPR3 expression [8]. Meanwhile, recent research indicated that BCYRN1 accelerated the proliferation and metastasis of cervical cancer through regulating miR-138 in vitro and in vivo [9]. And research from Ren et al. found that overexpression of BCYRN1 facilitated tumor progression and up-regulated EpCAM expression in gastric carcinoma [10]. Furthermore, BCYRN1 has been illustrated to be greatly expressed in HCC and its expression was actively connected with tumor-node-metastasis and unfavorable prognosis in HCC patients [11]. However, study on the potential molecular mechanisms of BCYRN1 in HCC was scarce. Therefore, our research mainly focused on the regulatory network of BCYRN1 in HCC.

Currently, a growing body of evidence indicated that both miRNA and lncRNA were involved in the pathological processes related with numerous human diseases. Therefore, lots of work has been done to explore the effects of miRNA on lncRNA functions and vice versa [12, 13]. It is well known that miRNAs directly bind to 3′UTR of target gene to degrade mRNA or suppress mRNA translation, thereby modulating gene transcription [14, 15]. Recently, extensive studies have revealed that numerous miRNAs are aberrantly expressed in HCC and take part in the development of HCC through modulating cell multiplication, survival and metastasis [16]. MiR-490-3p has been verified to suppress several cancers proliferation, metastasis, and progression including lung cancer, colorectal cancer, prostate cancer, esophageal squamous cell carcinoma and so on [17–20]. In addition, miR-490-3p has been confirmed to regulate cells growth and EMT in HCC cells by targeting ERGIC3 [21]. POU class 3 homeobox 2 (POU3F2), which belongs to the class III POU factors, has been reported to expedite tumor cells proliferation or even promote tumorigenesis [22–24]. To date, research on the regulation of miR-490-3p in HCC and its latent molecular mechanisms are still lacking. Our study intended to search the function and underlying mechanisms of BCYRN1, miR-490-3p, and POU3F2 in the progression of HCC, and expected to provide some useful molecules for targeted therapy of HCC.

Our data indicated that BCYRN1 was highly expressed in HCC samples and conduced to HCC cells growth and metastasis. Moreover, BCYRN1, acted as a competing endogenous RNA (ceRNA) of miR-490-3p, was verified by dual luciferase assay. Further experimentation insinuated that knockdown of BCYRN1 inhibited the enhancing impacts of miR-490-3p inhibitor or POU3F2 on HCC cells growth, invasion and migration abilities, and vice versa. All results explained the ceRNA network of BCYRN1/miR-490-3p/POU3F2 in HCC development, supplying newfangled targeted molecules for HCC treatment and prognosis.

## Materials and methods

### Data acquisition

Data arriving from The Cancer Genome Atlas (TCGA, https://cancergenome.nih.gov/) database containing 375 HCC patients and 50 normal were applied to assess the expression patterns of BCYRN1, miR-490-3p, and POU3F2 in HCC. 370 cases of HCC patients with correspondingly complete clinical information were applied to assess the associations between expression of BCYRN1, miR-490-3p, or POU3F2 and survival rates of HCC patients.

### Cell culture and transfection

Normal cell line L-02 and four HCC cell lines including HepG2, Huh7, Hep3B, and BEL-7402 were acquired from Chinese Academy of Science Cell Bank (Shanghai, China). Culture medium Roswell Park Memorial Institute (RPMI)-1640 including 10% fetal bovine serum, 100 U/mL penicillin, and 0.1 mg/mL streptomycin was applied to cultivate normal cell line L-02 in 5% CO_2_ incubator at 37 °C. And Dulbecco’s Modified Eagel Medium (DMEM) was used to incubated these HCC cell lines with the same condition.

GenePharma Co. (Shanghai, China) provided si-BCYRN1 (5′-CAGCTCTCAGGGAGGCTAAGA-3′), si-POU3F2 (5′- GTACCGGTGAAGCCTTTGTTGAACAAGTCTC-3′), si-con (5′- CACATGTAGGCATAGTCGTCACTCAGTG-3′), pcDNA3.1-BCYRN1, pcDNA3.1-POU3F2, miR-490-3p mimic/inhibitor and their negative control (NC). Lipofectamine 2000 (Invitrogen, USA) was utilized to transfect the aforementioned small fragments into cells to regulate the expression of BCYRN1, miR-490-3p, and POU3F2.

### RNA extraction and quantitative real-time PCR (qRT-PCR)

Whole RNA was extracted from the treated cells with TRIzol reagent (Invitrogen, USA). PrimeScript RT Reagent Kit was applied to reverse transcribed mRNA into cDNA and SYBR Premix Ex Taq II (TaKaRa, Japan) was used to perform qRT-PCR. GAPDH was served as an internal standard for detection of BCYRN1 and POU3F2. miRNA was reverse transcribed into cDNA by Mir-X™ miRNA First Strand Synthesis Kit and qRT-PCR was executed by SYBR PrimeScriptTM miRNA RT-PCT Kit (TaKaRa, Japan). U6 was regarded as an internal standard for detection of miR-490-3p. 2 ^− ΔΔCT^ method was employed to assess the relative expression of BCYRN1, miR-490-3p, and POU3F2.

### Dual luciferase reporter assay

Associations between BCYRN1 and miR-490-3p, as well as miR-490-3p and POU3F2 were affirmed by dual luciferase assay. Firstly, Wild Type (WT)-BCYRN1, Mutant (MUT)-BCYRN1, WT-POU3F2, or MUT-POU3F2 was cloned into luciferase vector pGL3. Subsequently, co-transfected luciferase vector and miR-490-3p mimic or miR-490-3p mimic NC into cells. The cells were transplanted into 96-well plate and cultured for 48 h. Eventually, lysed the cells and detected the luciferase activity by dual-luciferase report analysis system (Promega, Madison, WI, USA).

### Western bolt

After 24 h of cell transfection, radioimmunoprecipitation assay (RIPA) reagent with protease inhibitor was added to extract protein, protein concentration was determined by bicinchoninic acid (BCA) method and adjusted to be consistent. The protein (20 μg) was loaded into each hole of the vertical electrophoresis tank, isolated by SDS-polyacrylamide gel electrophoresis, and transferred onto polyvinylidene difluoride (PVDF) membranes. The membranes were sealed for 1 h with 5% defatted milk, incubated with primary antibody POU3F2 (Abcam, UK, ab243045, 1 μg/mL,) at 4 °C over 12 h, washed with tris buffered saline tween (TBST) for three times, and incubated with secondary antibody at about 25 °C for 2 h. Finally, electrochemiluminescence (ECL) reagent (Abbkine, China) was added to develop the signals and QUANTITY ONE software was used to scan the gray bands. GAPDH was regarded as an internal criterion.

### Cell proliferation assay

Cell proliferation ability was determined by CCK-8 reagent (Dojindo, Japan). The cells were implanted into 96-well plate with a standard of 1000 cells per well after transfection for 24 h. The cells were routinely cultured in a carbon dioxide incubator, and the cell activity was assessed every 24 h. Added 10 μL of CCK-8 reagent into each well and cultured for 2 h in a 37 °C incubator before every detection. The optical density (OD) values were calculated at 450 nm wavelength with a microplate reader, and a proliferation curve was drawn using GraphPad Prism 5.0.

### Clone formation assay

Cells in logarithmic phase were digested with trypsin and blown into single cell. Implanted single cell into a 60 mm dish containing 5 mL 37 °C pre-culture medium at the density of 500 cells per dish. The cells were cultivated at 37 °C with 5% CO_2_ and saturated humidity for about 10 days. As soon as visible clones appeared in per dish, the culture was terminated. Then, 4% paraformaldehyde and 0.1% crystal violet were used to fix and stain the cells. Finally, washed away the dye slowly with running water and dried in the air. Visually count the number of clones.

### Cell invasion and migration assays

Transwell chambers were applied to assess cells invasion and migration abilities. For invasion assay, 100 μL of Matrigel was pre-coated onto the upper chamber of the 24-well plate transwell chamber, and 500 μL of serum-free medium was loaded into lower chamber. Subsequently, 100 μL of cell suspension including 1 × 10^5^ cells was added into the upper chamber, and 500 μL of complete culture solution was filled into the lower chamber. Over night, removed the chamber, and the residual cells in the upper chamber were wiped off. After PBS cleaning, cells adhering to the lower chamber were fixed and stained. Finally, 5 fields were randomly selected under the microscope for observation and counting.

The steps of migration experiment were analogous to the invasion assay, except that the upper chamber was not required pre-coated with Matrigel.

### Statistical analysis

All data was disposed with SPSS16.0 and GraphPad prism 5.0. Difference between two groups was assessed by student’s t test, while comparison among multiple groups was detected with ANOVA analysis following Dunnett or Bonferroni post hoc test. The prognostic survival rate was detected by Kaplan–Meier method and log-rank test. In the prognostic analysis, the expression levels of BCYRN1, miR-490-3p, and POU3F2 were divided into high expression level and low expression level based on the median value. A P value less than 0.05 was significantly statistical.

## Results

### High expression of BCYRN1 was connected with undesirable survival rate of HCC patients

**Fig. 1:**
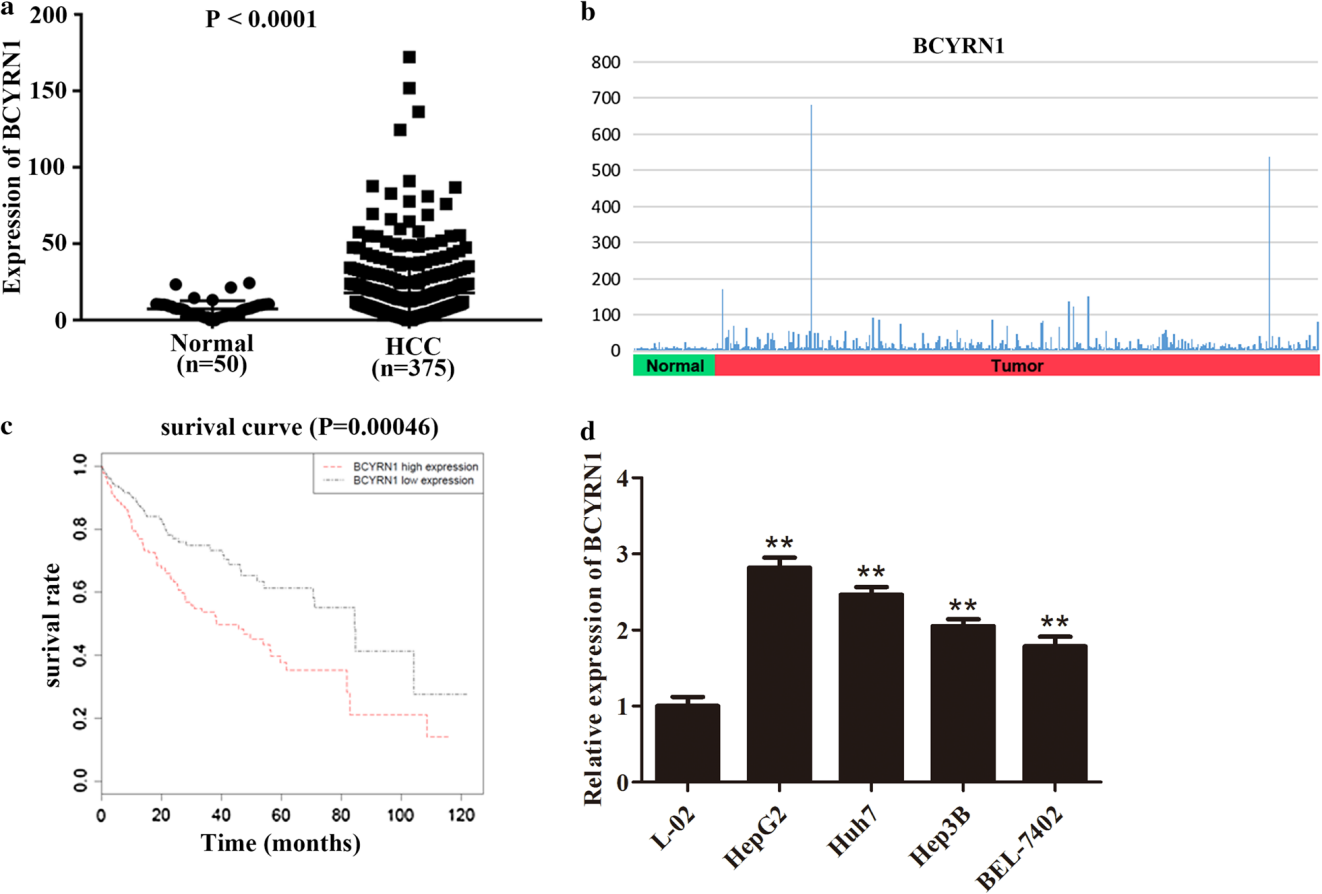
High expression of BCYRN1 was linked to unfavorable prognosis of patients in HCC. **a** The data from TCGA showed that BCYRN1 was obviously up-regulated in HCC patients in contrast with the normal, P < 0.0001. **b** The BCYRN1 expression distribution pattern was presented. **c** HCC patients with overexpression of BCYRN1 were more likely led to worse prognosis, P = 0.00046. **d** BCYRN1 was overexpressed in HCC cell lines including HepG2, Huh7, Hep3B, and BEL-7402 in contrast with the normal cell line L-02, **P < 0.01

In accordance with data from TGCA, BCYRN1 was found to be highly expressed in HCC patients (n = 375) in contrast with normal (n = 50, Fig. 1a, b, P < 0.0001). To explore the relationship between BCYRN1 expression and survival rate of HCC patients, 370 cases of patients with relatively integrated clinical data were applied for survival analysis using Kaplan–Meier method. The consequence exhibited that the expression level of BCYRN1 was closely linked to the survival rate of HCC patients. Overexpression of BCYRN1 led to low survival rate of HCC patients, while patients with lower expression of BCYRN1 had better survival rate (Fig. 1c, P = 0.00046). Subsequently, five cell lines were applied to explore the expression level of BCYRN1 by qRT-PCR. As exhibited in Fig. 1d, BCYRN1 was remarkably up-regulated in HCC cell lines in contrast with the control, and the expression was highest in HepG2 cells and lowest in BEL-7402 cells, so HepG2 and BEL-7402 were picked up for following experiments (P < 0.01). Whilst, these phenomena implied that BCYRN1 played a crucial role in HCC progression.

### Up-regulation of BCYRN1 contributed to HCC cells proliferation, invasion, and migration

**Fig. 2:**
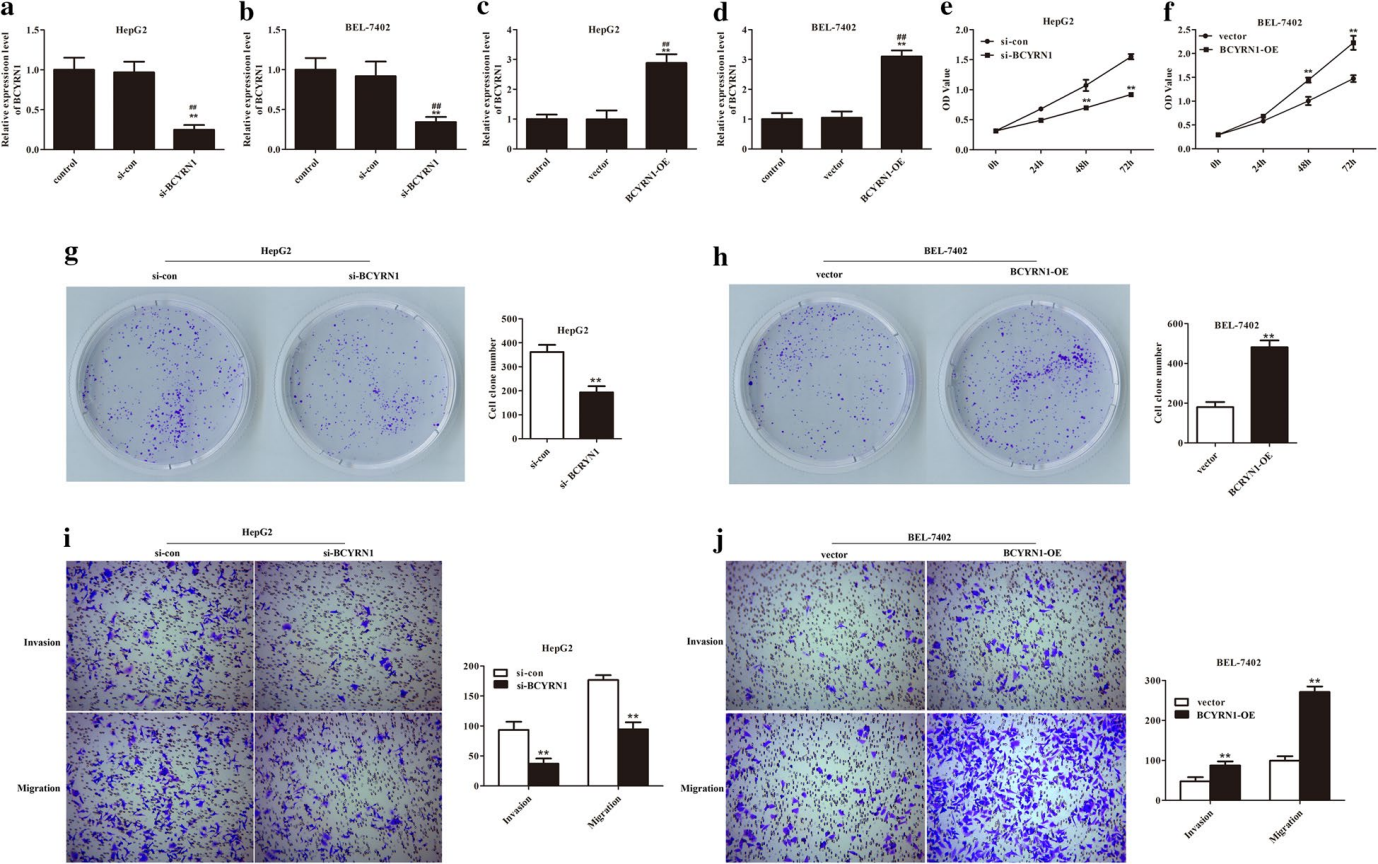
BCYRN1 strengthened HCC cells growth, invasion, and migration abilities. **a**, **b** The mRNA expression level of BCYRN1 was significantly reduced after transfected with si-BCYRN1, **P < 0.01 vs. control, ^##^P < 0.01 vs. si-con. **c**, **d** The mRNA expression level of BCYRN1 was obviously up-regulated after transfected with pcDNA3.1-BCYRN1, **P < 0.01 vs. control, ^##^P < 0.01 vs. vector. **e** The OD values of HepG2 cells were decreased after transfected with si-BCYRN1, **P < 0.01. **f** The OD values of BEL-7402 cells were increased after transfected with pcDNA3.1-BCYRN1, **P < 0.01. **g** The clone formation number of HepG2 cells was reduced after depletion of BCYRN1, **P < 0.01. **h** Overexpression of BCYRN1 elevated BEL-7402 cells colony forming ability, **P < 0.01. **i** The invaded and migrated cells number of HepG2 cells were significantly reduced after knockdown of BCYRN1, **P < 0.01. **j** Up-regulation of BCYRN1 enhanced BEL-7402 cells invasion and migration abilities, **P < 0.01

To study the function of BCYRN1 in HCC development, the downregulation and overexpression of BCYRN1 were firstly carried out. As presented in Fig. 2a, b, the expression of BCYRN1 was obviously decreased after transfected with si-BCYRN1 (P < 0.01). Whilst, after transfected with pcDNA3.1-BCYRN1, the expression of BCYRN1 was remarkably increased, supplying basis for subsequent experiments (Fig. 2c, d, P < 0.01).

Subsequently, CCK-8, clone formation, and transwell assays were utilized to detect the function of BCYRN1 on HCC cells. After silencing of BCYRN1, the OD values, clone formation number, and invaded and migrated cells number of HepG2 cells were significantly reduced compared with the control (Fig. 2e, g, i, P < 0.01). While, the OD values, clone formation number, and invaded and migrated cells number of BEL-7402 cells were clearly elevated compared with the control after overexpression of BCYRN1 (Fig. 2f, h, j, P < 0.01). Overall, these findings stated that BCYRN1 accelerated HCC cells proliferation and metastasis.

### Association between BCYRN1 and miR-490-3p in HCC was affirmed by dual luciferase assay

**Fig. 3:**
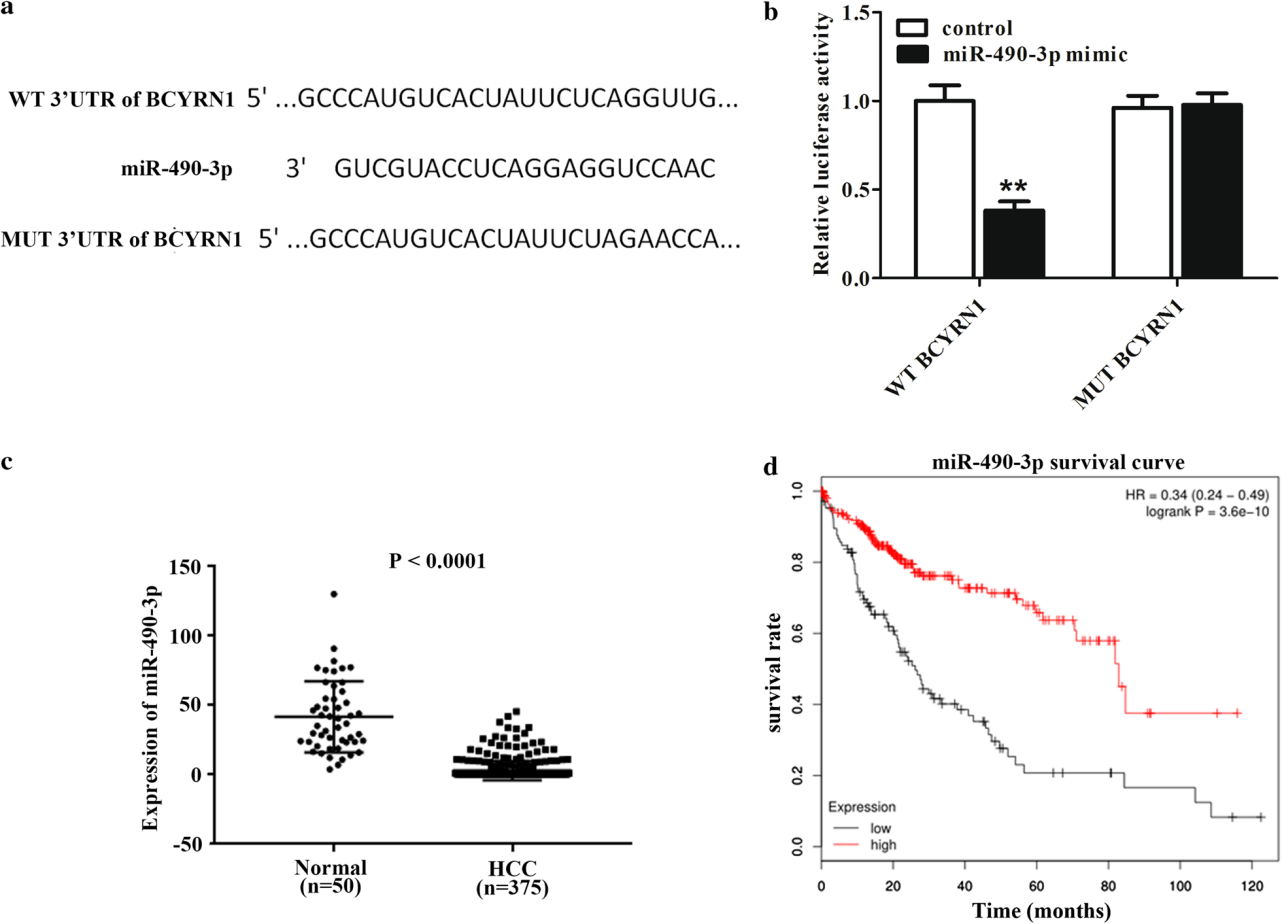
miR-490-3p was down-regulated in HCC patients and inversely modulated by BCYRN1. **a** The binding site between BCYRN1 and miR-490-3p was exhibited. **b** The luciferase activity was detected in WT-BCYRN1 and MUT-BCYRN1, **P < 0.01. **c** Low expression of miR-490-3p was presented in HCC patients, P < 0.0001. **d** HCC patients with lower expression of miR-490-3p easily resulted in unfavorable prognosis

On the basis of lncRNA target prediction website mircode and down-regulated miRNA genes in HCC patients, miR-490-3p was picked up as a target of BCYRN1. The binding sites between BCYRN1 and miR-490-3p were presented in Fig. 3a. Results from luciferase assay stated that miR-490-3p mimic transfection could decrease WT-BCYRN1 activity, but had almost no influence on MUT-BCYRN1 activity, which stated that miR-490-3p was a direct target of BCYRN1 (Fig. 3b, P < 0.01). To detect the connection between miR-490-3p and HCC, TCGA database including 375 HCC patients and 50 normal was employed. The data revealed that miR-490-3p was lower expressed in patients with HCC (Fig. 3c, P < 0.0001) and its low expression was closely linked to poor survival in HCC patients (Fig. 3d, P = 3.6e−0). All results stated that there was a targeted relationship between BCYRN1 and miR-490-3p, and lower expression of miR-490-3p was presented in patients with HCC, which was associated with HCC patients unfavorable prognosis.

### POU3F2, as a target of miR-490-3p, was overexpressed in HCC patients and upregulated by BCYRN1 through sponging miR-490-3p

**Fig. 4:**
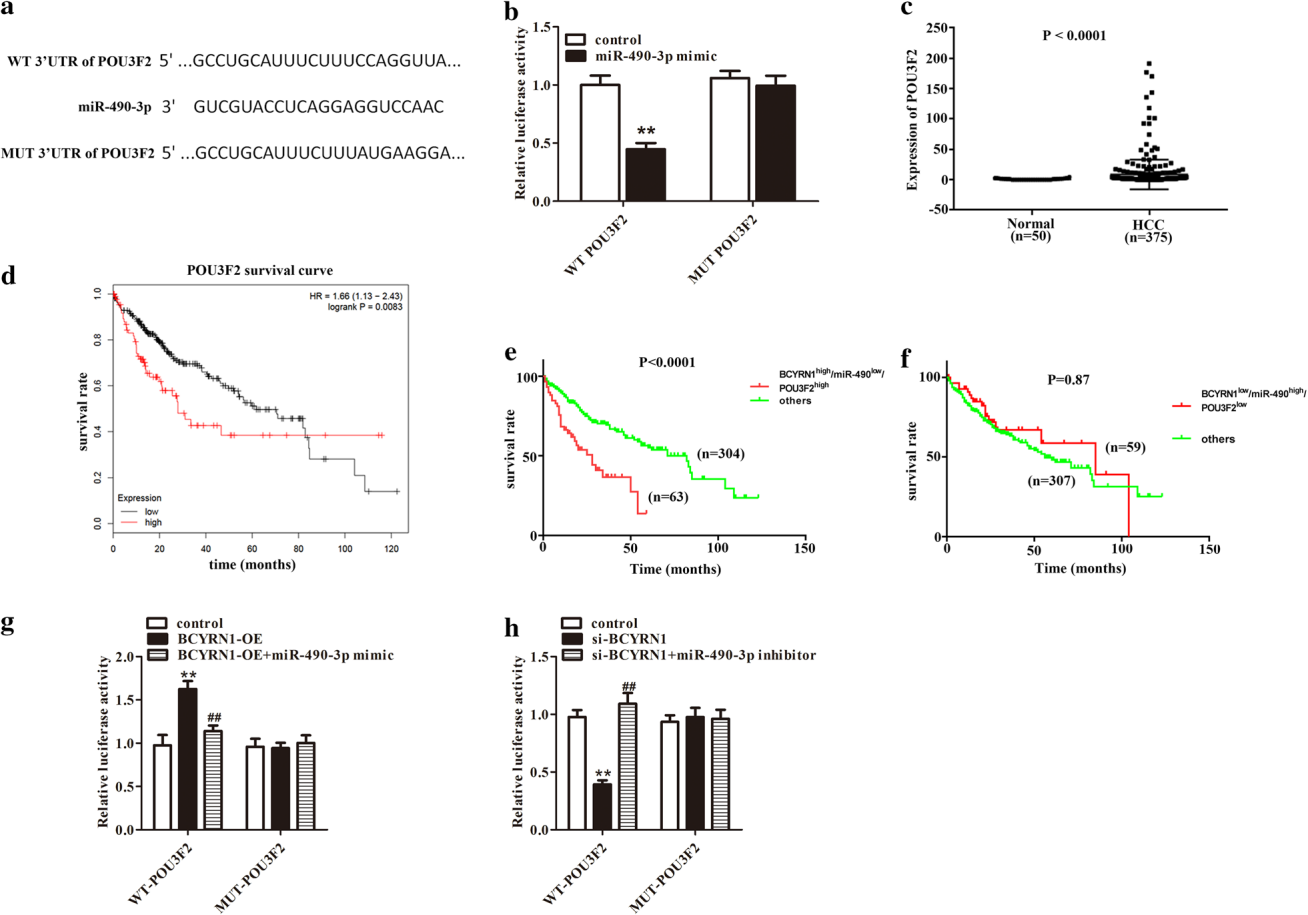
POU3F2, a downstream target of miR-490-3p, was over expressed in HCC patients. **a** POU3F2 was predicted as a target gene of miR-490-3p. **b** miR-490-3p mimic transfection reduced luciferase activity in WT-POU3F2 group, but had no effect on MUT-POU3F2 group, **P < 0.01. **c** POU3F2 was up-regulated in HCC patients, P < 0.0001. **d** High expression of POU3F2 in HCC patients was related with unfavorable prognosis. **e** The clinical relevance levels of BCYRN1^high^/miR-490-3p^low^/POU3F2^high^ in patients with HCC were presented, P < 0.0001. **f** The clinical relevance levels of BCYRN1^low^/miR-490-3p^high^/POU3F2^low^ in patients with HCC were exhibited, P = 0.87. **g** Upregulation of BCYRN1 elevated the luciferase activity of WT-POU3F2, and this phenomenon was inhibited after miR-490-3p overexpression, **P < 0.01 vs. control, ^##^P < 0.01 vs. BCYRN1-OE. **h** Knockdown of BCYRN1 reduced the luciferase activity of WT-POU3F2, and silencing of miR-490-3p attenuated this trend, **P < 0.01 vs. control, ^##^P < 0.01 vs. si-BCYRN1

Next, targetscan website was employed to predict target gene of miR-490-3p. In numerous putative targets of miR-490-3p, POU3F2 was selected due to a crucial pro-tumor effect in numerous cancers [25, 26]. The 3′UTR of POU3F2 (Fig. 4a) was fused to the luciferase coding region and transfected into cells with miR-490-3p mimic. The luciferase assay considered that POU3F2 was a direct target of miR-490-3p (Fig. 4b, P < 0.01). Meanwhile, the results from TCGA database indicated that POU3F2 was highly expressed in HCC patients (n = 375) compared to normal (n = 50), and its high expression was easily led to unfavorable prognosis in HCC patients (Fig. 4c, P < 0.0001, Fig. 4d, P = 0.0083). To detect clinical relevance of the correlation of LncRNA BCYRN1/miR-490-3p/POU3F2, the prognosis of HCC patients with LncRNA BCYRN1/miR-490-3p/POU3F2 co-expression were examined. Patients (n = 63) with BCYRN1^high^/miR-490-3p^low^/POU3F2^high^ showed unfavorable prognosis compared with others (n = 304, Fig. 4e, P < 0.0001). While, patients (n = 59) with BCYRN1^low^/miR-490-3p^high^/POU3F2^low^ exhibited an opposite trend, but the difference was not significant (Fig. 4f, P = 0.87). To further investigate associations among BCYRN1, miR-490-3p and POU3F2, dual luciferase assay was performed. Overexpression of BCYRN1 increased the luciferase activity of WT- POU3F2. But ectopic expression of miR-490-3p eliminated this upregulation (Fig. 4g, P < 0.01). Reciprocally, silencing of LncRNA BCYRN1 reduced the luciferase activity of WT-POU3F2, which was rescued by depletion of miR-490-3p (Fig. 4h, P < 0.01).

**Fig. 5:**
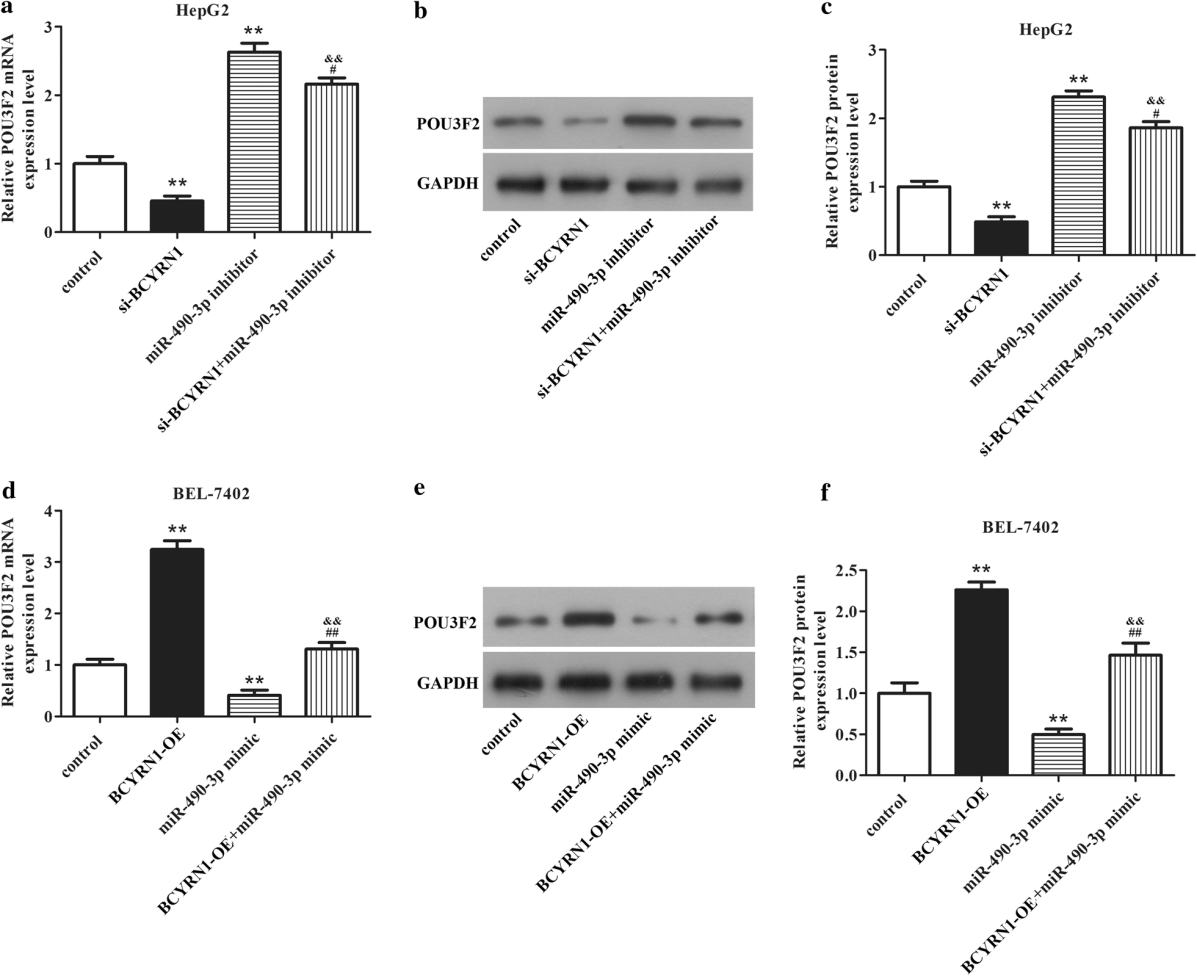
Associations among BCYRN1, miR-490-3p, and POU3F2 were assessed by western blot and qRT-PCR. The mRNA **a** and protein **b** expression of POU3F2 in HepG2 cells were evaluated after knockdown of BCYRN1, miR-490-3p, or BCYRN1 + miR-490-3p. **c** is statistical of **b**. **P < 0.01 vs. control, ^#^P < 0.01 vs. miR-490-3p inhibitor. The mRNA **d** and protein **e** expression of POU3F2 in BEL-7402 cells were determined after overexpression of BCYRN1, miR-490-3p, or BCYRN1 + miR-490-3p. **f** is statistical of **e**. **P < 0.01 vs. control, ^##^P < 0.01 vs. miR-490-3p mimic

Subsequently, qRT-PCR and western blot were executed to identify the associations among BCYRN1, miR-490-3p, and POU3F2. In HepG2 cells, the mRNA and protein expression levels of POU3F2 were obviously downregulated after knockdown of BCYRN1. But depletion of miR-490-3p increased the expression of POU3F2. However, knockdown of BCYRN1 and miR-490-3p could limit the promoting effect of miR-490-3p inhibitor on POU3F2 expression and the inhibitory effect of si-BCYRN1 on POU3F2 expression (Fig. 5a–c, P < 0.05). In BEL-7402 cells, the mRNA and protein expression levels of POU3F2 were remarkably elevated after overexpression of BCYRN1. However, upregulation of miR-490-3p significantly reduced POU3F2 expression. While, overexpression of BCYRN1 and miR-490-3p could suppress the inhibitory effect of miR-490-3p mimic on POU3F2 expression and the promotion effect of BCYRN1-OE on POU3F2 expression (Fig. 5d–f, P < 0.05). The data indicated that POU3F2 acted as a significant role in HCC cells progression, which was upregulated by BCYRN1 through sponging miR-490-3p.

### BCYRN1/miR-490-3p/POU3F2 formed a ceRNA network to regulate HCC cells growth and metastasis

**Fig. 6:**
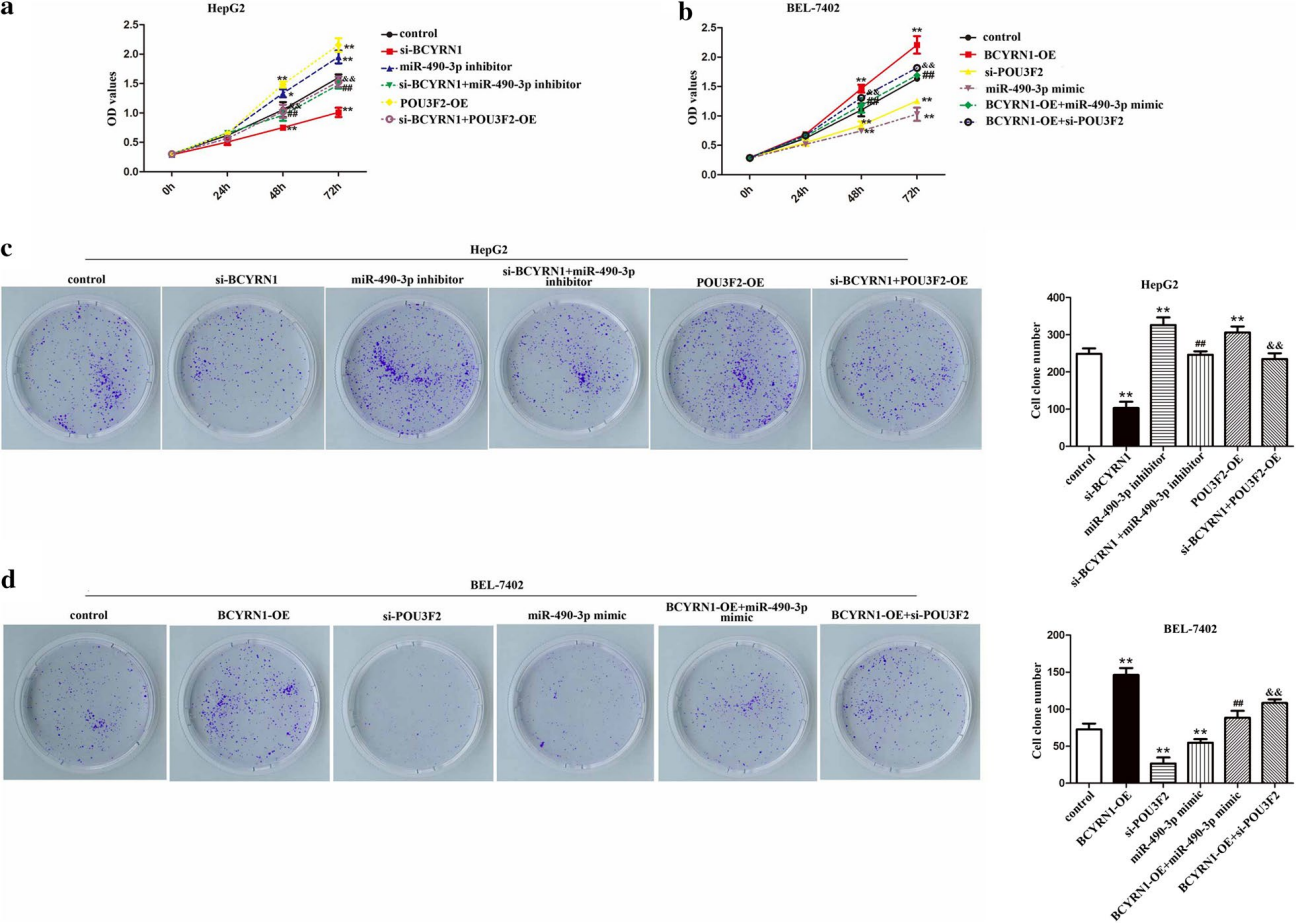
BCYRN1/miR-490-3p promoted HCC cells multiplication and colony formation by up-regulating of POU3F2. **a** The ascending OD values of HepG2 cells caused by miR-490-3p inhibitor or pcDNA3.1-POU3F2 were suppressed by depletion of BCYRN1, **P < 0.01 vs. control, ^##^P < 0.01 vs. miR-490-3p inhibitor, ^&&^P < 0.01 vs. POU3F2-OE. **b** The declining OD values of BEL-7402 induced by miR-490-3p mimic or si-POU3F2 were blocked by overexpression of BCYRN1, **P < 0.01 vs. control, ^##^P < 0.01 vs. miR-490-3p mimic, ^&&^P < 0.01 vs. si-POU3F2. **c** The increasing number of HepG2 cells clones induced by miR-490-3p inhibitor or pcDNA3.1-POU3F2 were inhibited by knockdown of BCYRN1, **P < 0.01 vs. control,^##^P < 0.01 vs. miR-490-3p inhibitor, ^&&^P < 0.01 vs. POU3F2-OE. **d** The decreasing number of BEL-7402 cells clones caused by miR-490-3p mimic or si-POU3F2 were impeded by up-regulation of BCYRN1, **P < 0.01 vs. control, ^##^P < 0.01 vs. miR-490-3p mimic, ^&&^P < 0.01 vs. si-POU3F2

**Fig. 7:**
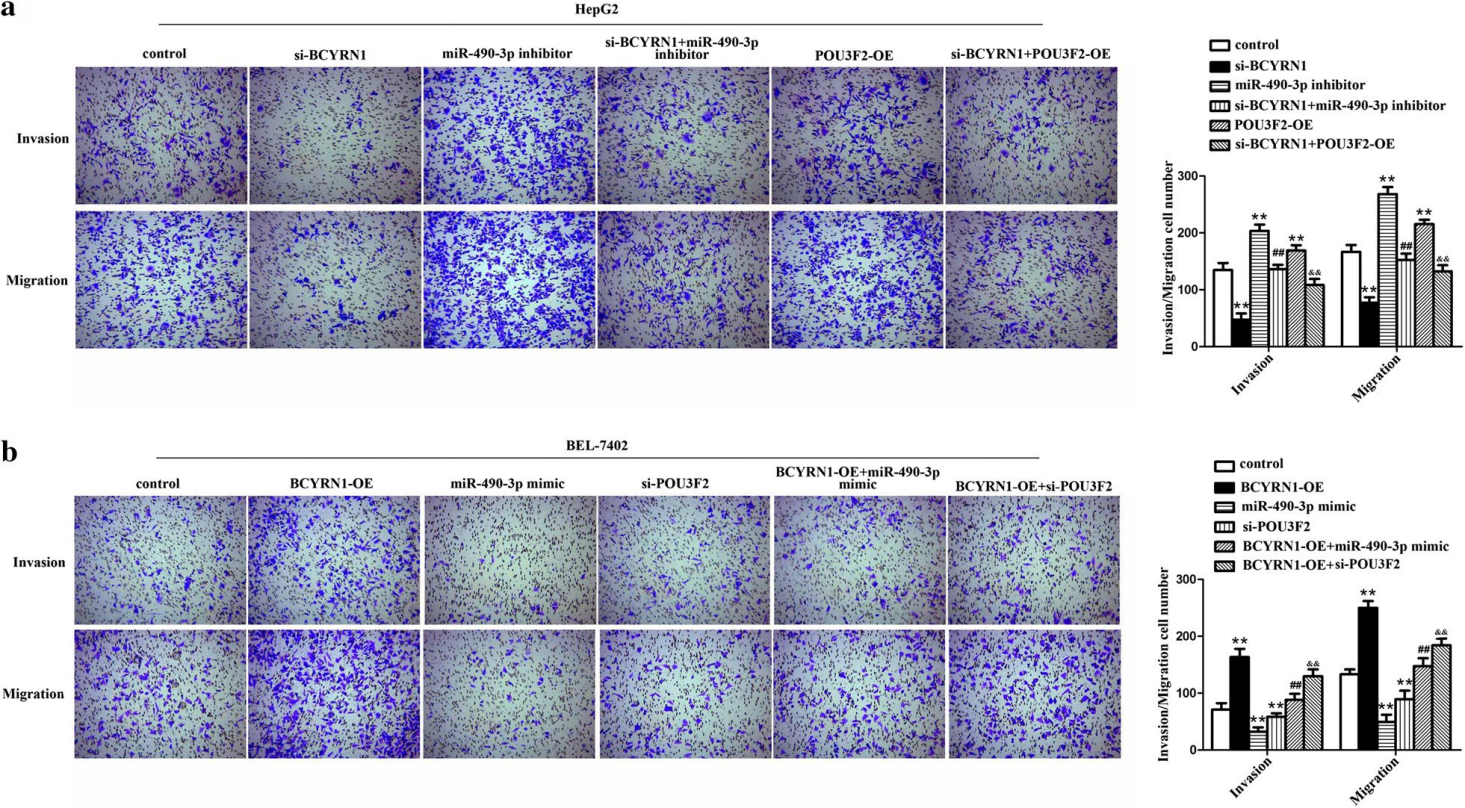
BCYRN1/miR-490-3p/POU3F2 formed a network to accelerate HCC cells invasion and migration. **a** The elevating of invaded and migrated HepG2 cells caused by miR-490-3p inhibitor or pcDNA3.1-POU3F2 were limited by silencing of BCYRN1, **P < 0.01 vs. control, ^##^P < 0.01 vs. miR-490-3p inhibitor, ^&&^P < 0.01 vs. POU3F2-OE. **b** The descending of invaded and migrated BEL-7402 cells induced by miR-490-3p mimic or si-POU3F2 were restricted by overexpression of BCYRN1, **P < 0.01 vs. control, ^##^P < 0.01 vs. miR-490-3p mimic, ^&&^P < 0.01 vs. si-POU3F2

To search function of BCYRN1/miR-490-3p/POU3F2 on HCC cells behavior, rescue assays were executed. The data showed that the OD values, clone formation number, invading and migrating cells number of HepG2 cells were elevated after transfected with miR-490-3p inhibitor or pcDNA3.1-POU3F2. However, knockdown of BCYRN1 could suppress the promoting impacts of miR-490-3p inhibitor or POU3F2 on HepG2 cells growth and metastasis (Figs. 6a, c, 7a, P < 0.01). Whilst, in BEL-7402 cells, the OD values, clone formation number, invading and migrating cells number were significantly reduced after treated with miR-490-3p mimic or si-POU3F2. But overexpression of BCYRN1 could alleviate the inhibitory effects of miR-490-3p mimic or si-POU3F2 on BEL-7402 cells growth and metastasis (Figs. 6b, d, 7b, P < 0.01). The whole data hinted that BCYRN1 upregulated POU3F2 expression through sponging miR-490-3p to promote HCC cells proliferation, invasion, and migration abilities.

## Discussion

It has been well demonstrated that several lncRNAs played vital roles in the modulation of HCC cells growth, invasion, and migration [27, 28]. For example, BCYRN1 has been identified to promote tumor growth and regulate metastasis in some cancers, such as non-small-cell lung cancer, colorectal cancer and so on [7, 8]. In our study, high expression of BCYRN1 has been presented in HCC patients and cells. And the survival rate of HCC patients with overexpression of BCYRN1 was remarkably less than that of HCC patients with lower BCYRN1 expression, suggesting that BCYRN1 overexpression was linked to unfavorable prognosis in HCC patients. So, our study initially explored the function and potential molecular mechanism of BCYRN1 in the progression of HCC.

According to numerous literature records, lncRNAs have played crucial roles in HCC development and progression. For example, lncRNA HANR has been identified to expedite tumorigenesis and increase of chemoresistance in HCC [29]. Research from Chen et al. stated that lncRNA CDKN2BAS was connected with poor survival rate in patients with HCC and promoted metastasis through miR-153-5p/ARHGAP18 [30]. It has been observed that lncRNA WDR26 limited the multiplication and metastasis of HCC cells by interacting with SIX3 [31]. In our research, BCYRN1 was overexpressed in HCC patients and its high expression was linked to unfavorable prognosis in HCC patients. In addition, overexpression of BCYRN1 contributed to BEL-7402 cells proliferation, invasion, and migration. While, knockdown of BCYRN1 inhibited HepG2 cells growth and metastasis.

Previous research stated that lncRNAs modulated target gene expression by variant mechanisms in diverse cancer cells [31]. It is worth noting that lncRNAs can serve as ceRNAs, also called miRNA sponge, limit miRNAs expression to regulate the derepression of miRNAs targets [32, 33]. Through bioinformatics software and luciferase assay, we discovered that BCYRN1 directly interacted with miR-490-3p to function in HCC development. MiR-490-3p, as a tumor suppressor, has attracted extensive attention. A recent study stated that miR-490-3p limited the tumorigenesis and development of ovarian epithelial carcinoma [34]. Research from Xu et al. exhibited that miR-490-3p limited colorectal cancer metastasis by regulating TGFβR1 [18]. In addition, a report has been illustrated that miR-490-3p suppressed the multiplication and metastasis of esophageal squamous cell carcinoma via modulation of HMGA2 [20]. In our study, down-regulation of miR-490-3p has been observed in HCC patients and its low expression was connected with unfavorable prognosis in HCC patients. And knockdown of miR-490-3p could promote HCC cells proliferation, invasion, and migration abilities. While, depletion of BCYRN1 limited the promoting impacts of miR-490-3p inhibitor on HCC cells growth and metastasis. The results stated that miR-490-3p was negatively controlled by BCYRN1 to suppress HCC cells development.

It is well known that miRNAs functioned in various diseases by interacting with their target genes [35]. Luckily, POU3F2 was selected as a target of miR-490-3p due to prediction software analysis and literature review. POU3F2, which belongs to the class III POU factors, also called Brn2 or N-Oct3, has been reported to be closely associated with cell proliferation or even elevated the potential for metastasis [22–24]. Brn2 was revealed to inhibit apoptosis and reprogram DNA damage repair, and was connected with a high somatic mutation burden in melanoma [36]. It has been observed that POU3F2 conduced to cellular responses against oxaliplatin in human colon cancer cells by regulating tNOX [37]. In our study, POU3F2 was demonstrated to be highly expressed in HCC patients and related with poor survival rate of HCC patients. Whilst, overexpression of POU3F2 enhanced HCC cells growth and metastasis. However, silencing of BCYRN1 attenuated the promoting effects of POU3F2 on HCC cells proliferation, invasion, and migration capacities. These findings suggested that POU3F2 was positively regulated by BCYRN1 to promote the progression of HCC cells.

## Conclusion

In general, dissimilarly expression patterns of BCYRN1, miR-490-3p, and POU3F2 have been identified in HCC patients, which were related to the poor prognosis of patients. Our outcomes suggested that BCYRN1, miR-490-3p, and POU3F2 formed a ceRNA mechanism to modulate the occurrence and progression of HCC, providing a theoretical basis for searching neoteric target molecules for the treatment of HCC.

## Data Availability

Not applicable.

## Abbreviations

BCYRN1: Brain Cytoplasmic RNA 1
HCC: hepatocellular carcinoma
POU3F2: POU class 3 homeobox 2
TCGA: The Cancer Genome Atlas
qRT-PCR: quantitative real time polymerase chain reaction
CCK-8: cell counting kit-8
ceRNA: competing endogenous RNA
RIPA: radioimmunoprecipitation
BCA: bicinchoninic acid
ECL: electro chemiluminescence
GAPDH: Glyceraldehyde-3-Phosphate Dehyhrogenase
PBS: phosphate buffer solution
OD: optical density
ANOVA: one-way analysis of variance
